# Gene network analysis reveals a role for striatal glutamatergic receptors in dysregulated risk-assessment behavior of autism mouse models

**DOI:** 10.1038/s41398-019-0584-5

**Published:** 2019-10-17

**Authors:** Oded Oron, Dmitriy Getselter, Shahar Shohat, Eli Reuveni, Iva Lukic, Sagiv Shifman, Evan Elliott

**Affiliations:** 1 Molecular and Behavioral Neurosciences Lab, Bar-Ilan University Faculty of Medicine, 1311502 Safed, Israel; 2 Department of Genetics, The Alexander Silberman Institute of Life Sciences, The Hebrew University of Jerusalem, 9190501 Jerusalem, Israel

**Keywords:** Molecular neuroscience, Genomics

## Abstract

Autism spectrum disorder (ASD) presents a wide, and often varied, behavioral phenotype. Improper assessment of risks has been reported among individuals diagnosed with ASD. Improper assessment of risks may lead to increased accidents and self-injury, also reported among individuals diagnosed with ASD. However, there is little knowledge of the molecular underpinnings of the impaired risk-assessment phenotype. In this study, we have identified impaired risk-assessment activity in multiple male ASD mouse models. By performing network-based analysis of striatal whole transcriptome data from each of these ASD models, we have identified a cluster of glutamate receptor-associated genes that correlate with the risk-assessment phenotype. Furthermore, pharmacological inhibition of striatal glutamatergic receptors was able to mimic the dysregulation in risk-assessment. Therefore, this study has identified a molecular mechanism that may underlie risk-assessment dysregulation in ASD.

## Introduction

Autism spectrum disorder (ASD) is defined by the presence of social-communicational deficits and repetitive behavior/restricted interests. A major burden for individuals diagnosed with ASD is an increase in self-injury and a higher rate of mortality due to unintentional injury such as drowning and suffocation^1^. Several studies have outlined sub-optimal risk assessment in individuals with ASD, partially explaining the rate of higher immortality and self-injury^2–6^. However, there is little knowledge of brain mechanisms involved in dysregulation of risk assessment in individuals with ASD.

One of the central brain regions responsible for risk-related decision-making processes is the striatum^7^. For example, striatal brain activity has been associated with high-risk gambling choices: when presented with choices of high gaining prospects, multiple areas including the dorsal and ventral striatum were highly active. In contrast, when presented with greater loss potential, there was a decrease in striatal activity^8^. Additionally, risk-assessment and risk-taking behaviors have been correlated with dopamine clearance in the dorsal striatum of rats^9^. This evidence suggests that the striatum has a role in risk-related behaviors.

Several lines of evidence have suggested a role for striatal dysfunction in the neuropathology of ASD. Volume and functional connectivity increase of multiple components of the dorsal striatum have been determined in patients diagnosed with ASD^10–12^. Previous studies have shown that individuals diagnosed with ASD have poor performance in the delayed discounting task, which is used to evaluate decision-making processes^13,14^. Interestingly, reduced delayed discounting in individuals with ASD was correlated with diminished activity in the basal ganglia, including the dorsal striatum^13,15^. Taken together, an in-depth study of risk-assessment behavior in ASD, and the role the striatum, is warranted.

ASD has a heritability rate of 64–91%^16^, suggesting that a genetic component is partly responsible for the disorder’s development. Therefore, discovering autism-associated genes should lead to an understanding of the molecular pathways involved in autism-related behaviors^17^. Thus far, variations in over 900 genes, or copy number variations (CNVs), have been associated with ASD development while a small subset of these variations are found to be very strongly associated with ASD or responsible for syndromic ASDs^18,19^. Many genetic mouse models have been developed that harbor genetic variations associated with ASD development. Comparing between these models, both behaviorally and molecularly, is a promising method to understand what molecular pathways regulate certain common behaviors in ASD^20^. For example, Kloth et al. studied five ASD mouse models to determine cerebellar-based sensory deficits therefore illuminating a possible mechanism for ASD-related dysfunction in sensation^21^. An additional study compared the neuroanatomy of 26 different ASD mouse models and determined consistent volumetric changes in the parieto-temporal lobe, cerebellar cortex, hypothalamus, and striatum^22^. In addition, a recent study examined a specific subset of cortical neurons which appear to be responsible for social deficits in three separate ASD mouse models^23^. These examples reveal that parallel biological and molecular characterization of multiple ASD models is beginning to uncover important biological features that are responsible for behavioral phenotypes. Nonetheless, there have been few studies into the molecular mechanisms underlying dysfunction of risk-assessment phenotypes in ASD.

In the current study, we compared behavioral and molecular characteristics of male mice in four ASD mouse models. Three of these models bear genetic deletions in genes highly associated with ASD, including the *SHANK3b* KO (exons 13–16), the *CASPR2* KO and Chr16p11.2df mouse models. In addition, we used the BTBR mouse, a strain that displays the core behavioral characteristics of ASD^24–26^.

Interestingly, all of these models have previously shown changes in striatal physiology and function. The *SHANK3b* KO models shows increased neuronal arborization, decreased spine density and thinner post synaptic densities (PSDs) in striatal MSNs^24^ The *CASPR2* KO model shows alterations in striatal interneurons^25,27^ the Chr16p11.2df model shows reduced striatal volume and increased striatal MSNs’ mini excitatory post synaptic currents (mEPSCs)^26^ and finally, changes in striatal dopamine and serotonin levels were observed in BTBR mice^28^. Considering the potential role the dorsal striatum has in risk-assessment behavior, we decided to test if risk-assessment is affected in the models, and what striatal molecular changes drive this behavior.

We determined dysfunctional risk-assessment behaviors in multiple ASD models in elevated plus maze (EPM) and dark/light (DL) test, two classic paradigms that test the conflict between exploration and safety. Striatal transcriptome analysis determined a glutamatergic protein interaction network that correlate with the dysfunctional risk-assessment behavior. Pharmacologic inhibition of this glutamatergic pathway in the dorsal striatum of wild type mice mimicked the risk-assessment phenotype and exacerbated this behavior in the *SHANK3b* KO and the *CASPR2* KO models. Therefore, we have identified a region-specific signaling pathway that may mediate risk-assessment behaviors in ASD.

## Materials and methods

### Animals

Mice were housed according to Federation of Laboratory Animal Science Associations (FELASA) guidelines. All mice were bred and maintained in a vivarium at 22C in a 12-h light/dark cycle, with food and water available ad libitum. Four ASD models were used in the experiments: The BTBR T+tf/J strain, which was provided by Dr. Tali Kimchi (Weizmann Institute of Science), the *CASPR2* KO line, which was provided by Prof. Elior Peles (Weizmann Institute of Science), the *SHANK3b* KO line, which was purchased from Jackson Laboratories, and the Ch16p11.2df line, which was provided from Prof. Alea Mills (Cold Spring Harbor Laboratories) and C57BL/6J were purchased from Jackson Laboratories. *SHANK3b KO* and *CASPR2* KO and wild type littermate mice were produced through crosses of heterozygote males and females. From the Chr16p11.2df mice and wild type littermates were produced through crosses of deletion males and wild type females. The genetic background for the *CASPR2* and *SHANK3b* mouse lines are C57BL/6J. For the Chr16p11.2df, upon arrival the background strain was a hybrid of C57BL/6J and C57BL/6N, which were backcrossed with C57BL/6J for four generations before experimentation. Experiments were performed with 8–10-week-old male mice. All experimental protocols were approved by the Animal Care and Use Committee of Bar Ilan University.

### Genotyping

To determine the genotypes of the Shank3, Caspr2, and Chr16p11.2 mice, DNA was extracted from ear samples notched at the time of weaning using the Kapa mouse genotyping kit. The following primers were used to determine Shank3 mice genotype: common Fw 5′-GAGCTCTACTCCCTTAGGACTT-3′; Rv mutatnt 5′-TCAGGGTTATTGTCTCATGAGC-3′ (~330 bp) and for wild type: Rv 5′- TCCCCCTTTCACTGGACACCC-3′ (~250 bp). To determine Caspr2 mice genotype: Fw mutant 5′-TTGGGTGGAGAGGCTATTCGGCTATG-3′ (~1000 bp); Fw wild type 5′- TCAGAGTTGATACCCGAGCGCC-3′ (~400 bp); common Rv 5′- TGCTGCTGCCAGCCCAGGAACTGG-3′. To determine Chr16p11.2df mice genotype: Fw 5′-TGCTGCTGCCAGCCCAGGAACTGG-3′; Rv 5′-CCAGTTTCACTAATGACACA-3′ (~2700 bp).

### Behavioral experiments

In all behavioral experiments, mice were housed in a reverse cycle room. All mice were randomly distributed into their experimental groups, and experimenters were blind to genotype at the time of the experiment. Analysis was also blind to genotype and treatment, as it was done by automatic software. The behavioral experiments schedule was designed so that the mice were initially exposed to the less anxiogenic paradigm (OF), then to Dark Light Test, and finally to the most anxiogenic test (EPM). 1 day of rest was allowed for the mice between behavioral experiments. While multiple testing on the same animals may affect results on subsequent tests, a previous study has found that a 1 day rest period is adequate after the open field (OF), dark light, and EPM tests^29^. Animals were excluded from experiments if they displayed sickness and injuries or showed little locomotion. Several mice were excluded specifically from the glutamate inhibition experiments if they would not successfully awaken from anesthesia post NBQX or vehicle infusion for at least 20 min. Mice were also excluded if the cannulae separated from their head. One *CASPR2* KO infused with NBQX was excluded due to lack of movement after infusion, and one C57BL/6J was removed due to cannulae separation. The experiments were recorded with the Panasonic WV-CL930 camera, and with the aid of the Ganz IR 50/50 Infrared panel, to enhance the detection of the mice. Mouse positioning and movement were analyzed by the Ethovision XT 10 (Noldus, Wageningen, The Netherlands) software.

### Rotarod

Rotarod tests were used to study the locomotor activity. The test was conducted using an accelerating Rotarod (Med Associates, St. Albans, VT). The speed of the Rotarod was set to 40 r.p.m. The amount of time each mouse spent on the rod was measured. The latency to fall was recorded with a 300 s cutoff time. Latency to fall average was calculated from three trials per mouse.

### Open field

Mice were placed in a corner of a square arena made from a Non-Glare Perspex (50 × 50 cm) which was illuminated at 40 lx. During the 10-min trial we measured total distance moved to evaluate locomotion and time in center (25 × 25 cm) to evaluate anxiety-like behavior.

### Dark/light test

The light–dark transfer test consists of a polyvinyl chloride box divided into a black dark compartment (14 × 27 cm) and a white 1200 lx illuminated light compartment (30 × 27 cm) connected by a small passage. The mouse was introduced to the dark compartment at the beginning of the 5 min trial and allowed to travel freely among the compartments. During the 5-min trial, time spent in the lighted zone and entry zone were measured to evaluate anxiety-like behavior and risk-assessing behavior.

### Elevated plus maze

The elevated plus-maze apparatus consists of a gray polyvinyl chloride maze, comprised of a central part (5 × 5 cm), two opposing open arms (30.5 × 5 cm), and two opposing closed arms (30.5 × 5 × 10 cm). The apparatus was elevated at a height of one meter and the open arms were illuminated with 15 lx. Mice were placed in the center, facing an open arm to initiate a 5 min session test. During the 5-min trial, time spent in open arms, entry zone to open arms and center was measured to evaluate anxiety-like behavior and risk-assessing behavior. Automatic stretch-attend postures were verified manually on two separate experiments. Manual scoring was performed blind to the group’s genotype or treatment (Supplementary Fig. S1).

### Cannula implantation

Mice were administered with pain reliever Buprenorphine (0.03 mg/ml) IP an hour before surgery, and then sedated with Isoflurane throughout the surgery using an electronic pump (NorVap Ltd., Skipton, UK). Mice were fixed in a stereotactic frame (Stoelting Co., Wooddale, IL). Two holes were drilled to expose brain tissue using a microdrill (RWD Life Science Inc., San Diego, CA). Then, a guide cannula of 0.64 mm OD (RWD-life sciences Inc.) was placed bilaterally, at the following co-ordinates AP = 0.14; DV = −3.5; ML = ±2 from bregma. Dummy cannulas (which extend 0.1 mm further than the guide cannula) were inserted into each guide cannula to prevent clogging. The cannulas were then anchored to the skull using C&B-Metabond® Quick! Luting Cement (Parkell Inc., Edgewood, NY) and further augmented using dental cement. The mice were allowed to recover for at least a week before behavioral experiments commenced.

### NBQX administration

NBQX disodium salt hydrate (Sigma-Aldrich, Rehovot, Israel) was resuspended in Hank’s balanced salt solution (HBSS) (Biological Industries, Kibbutz Beit-haemek, Israel) to a concentration of 3 µg/µl. Each mouse was infused bilaterally with either 3 µg of the α-amino-3-hydroxy-5-methyl-4-isoxazolepropionic acid (AMPA) and Kainate antagonist NBQX disodium salt hydrate or HBSS as control at a rate of 0.250 µl/1 min. Infusions were made through an injection cannula (RWD-life sciences Inc.) that was attached by polyethylene tube to a Hamilton microsyringe. The injection cannula was left in place for 1 min after the infusion was completed to allow for optimal absorbance of the injected liquid and the animals were tested within 10–20 min after infusion completion.

### Brain sample dissection

Brain samples were removed from mice that had not been subjected to any behavioral testing and were kept at normal light cycle facilities (not reverse light cycle). The entire mouse brain was removed at ~12:00 p.m. (light cycle is 7:00 a.m. to 7:00 p.m.) and placed in an adult mouse brain matrix (Zivic Industries, Pittsburgh, USA). Brain slices (bregma −0.58–1.53) were removed and dorsal striatum was obtained by using a 13-gauge biopsy punch needle (VGC, New Delhi, India). Brain samples were frozen with dry ice and kept in −80° until mRNA extraction.

### RNA sequencing and differential expression analysis

RNA was extracted from dorsal striatum samples with the RNeasy mini kit (Qiagen, Hilden, Germany). RNA Integrities were measured with the 2100 Bioanalyzer and evaluated with the TapeStation Software (A.01.03). All samples had RNA integrity of >8.4. Libraries for RNA-seq were prepared with the TruSeq RNA Library Prep kit v2 (Illumina, San Diego, CA). RNA was extracted from six mice per experimental group and two biological duplicates were pooled together to form a library, concluding with three libraries per experimental group (mouse line). Sequencing took place at the Technion Genome Center, Haifa with the Illumina HiSeq 2500. Fastq files are available at GEO under the accession number GSE138539. Since we received at least 35 million reads for each sample (35–60 million per sample), we subsampled each sample to receive 35 million reads, and then mapped to the Mus Musculus reference genome (mm9) using the Tophat2 software (release Tophat2.0.12). For differential expression analysis, we used the script cuffdiff v2.2.1 according to the published protocol^30^. All default parameters were used, except for modifying to unstranded sequencing. Statistics for each gene in each of the differential expression analysis, including FDR corrected *P*-values are found in Supplementary Tables 1–4.

### RT-PCR

Real-time PCR was performed on an ABI ViiA™ 7 RealTime PCR detection system in 10 μl volume containing FastStart Universal SYBR Green Master (Roche, Basel, Switzerland) and primers (Supplementary Table 5) at a concentration of 0.5 μM each. 10 ng of cDNA was dispersed in each well, and all samples were tested in triplicates. PCR program consists of 15-min activation phase at 95 °C, followed by 40 cycles at the following temperatures: 10 s of 94 °C, 30 s of 60 °C. Real-time PCR data were normalized to the housekeeping gene HPRT.

### Weighted gene coexpression network analysis (WGCNA)

The WGCNA R software package was applied to the normalized FPKM data^31^. Genes were excluded from the analysis if they were not expressed more than 10 samples. Briefly, pairwise bicor correlations between expression values were used to build a signed network. A soft threshold of 10 was used, as defined by the scale-free topology criterion. Subsequently, average linkage hierarchical clustering coupled with a topological overlap matrix (TOM)-based dissimilarity measure, were employed to construct a dendogram of the network whose branches, defined by the “dynamic tree cut” function, corresponded to single modules. The threshold to merge closely related modules was set at minimum height of 30. Each module was assigned a color, and a module eigengene (ME) corresponding to its first principal component, was calculated. In order to test the association between the modules and the phenotypic traits, we calculated the Pearson correlation between the MEs and the traits. For each trait the resulting *P*-values were corrected for multiple testing with the Benjamini and Hochberg false discovery rate (FDR) procedure.

The ME can be correlated to any sample trait (e.g., behaviors expressed by the animal models) to assess the significance of module–trait association (eigengene significance). For each gene, WGCNA defines the module membership (MM) that is the correlation between its expression values across samples and the ME. In addition, to incorporate external information into the co-expression network, we commutated the gene significance, that is the absolute value of correlation between each gene and a given trait. In our case, the gene significance is the correlation between the expression of each gene and a mouse behavior. Information about MM of each gene can be found in Supplementary Table 6.

### Protein–protein interaction (PPI) network analysis

Initially, gene lists from each WGCNA module were loaded onto the STRING database, and high confidence protein interactions (0.7) were searched for. Then, the yielded networks were further analyzed in Cytoscape (version 3.2.1): the network topography was based on the confidence score calculated by STRING. The Cytoscape MCODE v1.5 application was used to reveal strongly interconnected subnetworks. Additionally, BetweennessCentrality (calculates the shortest path a node creates between two other nodes) and Degree (which counts the number of interactions each node has) were used to locate central hubs which could be targeted for further experimentation.

### Synapse extraction

Synaptosomal extraction was performed using the Syn-PER synaptic protein extraction reagent (Thermo Scientific, Waltham, MA). Each frozen sample was gently homogenized, on ice, using a dounce in 150 µl of Syn-PER reagent and 1 µl of Halt Protease and Phosphatase Inhibitor Cocktail (Thermo Scientific) until no visible tissue was seen. The homogenate was centrifuged at 1200×*g* for 10 min at 4 °C, and the supernatant was removed and centrifuged again at 15,000×*g* for 20 min at 4 °C. The supernatant was saved for cytosolic fraction analysis, and the synaptosomal pellet was resuspended in 40 µl of Syn-PER reagent and Halt inhibitor cocktail master mix.

### Western blot

Protein concentrations were determined using Pierce BCA Protein Assay Kit (Thermo Scientific). Samples (30 μg) were subjected to SDS–PAGE and transferred onto a nitrocellulose membrane. The membrane was blocked for 1 h in 1XTBST containing 5% BSA followed by overnight incubation with a primary antibody in 5% BSA. The primary antibodies used were the following: anti-Gria4 1:5000 (Abcam, Cambridge, UK), anti-Grik1 1:500 (Santa Cruz Biotechnology, Dallas, TX) and anti-actin 1:200 (Santa Cruz Biotechnology). Next day, the membrane was washed with 1XTBST and incubated with secondary antibodies (Li-Core, Bad Homburg, Germany) at a ratio of 1:10,000 for 1.5 h. Membranes was then scanned on the LI-COR Odyssey scanner. Protein signals were measured with the Image Studio v2.0 software.

### Statistical analysis

All behavioral and molecular experiments (i.e., RTpcr and WB) were analyzed with SPSS V21 (IBM corp.). Levene’s test was used to test for equality of variance and Shapiro–Wilk for normal distribution. Two-tailed independent *t*-test was used for groups with equal variance and normal distribution. Mann–Whitney *U*-test was used to compare groups which did not display normal distribution, and Welch’s *t*-test was used when variance was unequal. Data are presented as mean ± SEM. Correlation between WGCNA modules and mouse traits, was evaluated with Pearson’s *r* correlation. To evaluate for significant correlations, *p* values were calculated, and FDR corrected for multiple comparisons.

## Results

### Atypical risk-assessment and anxiolytic behaviors observed in ASD mouse models

**Fig. 1:**
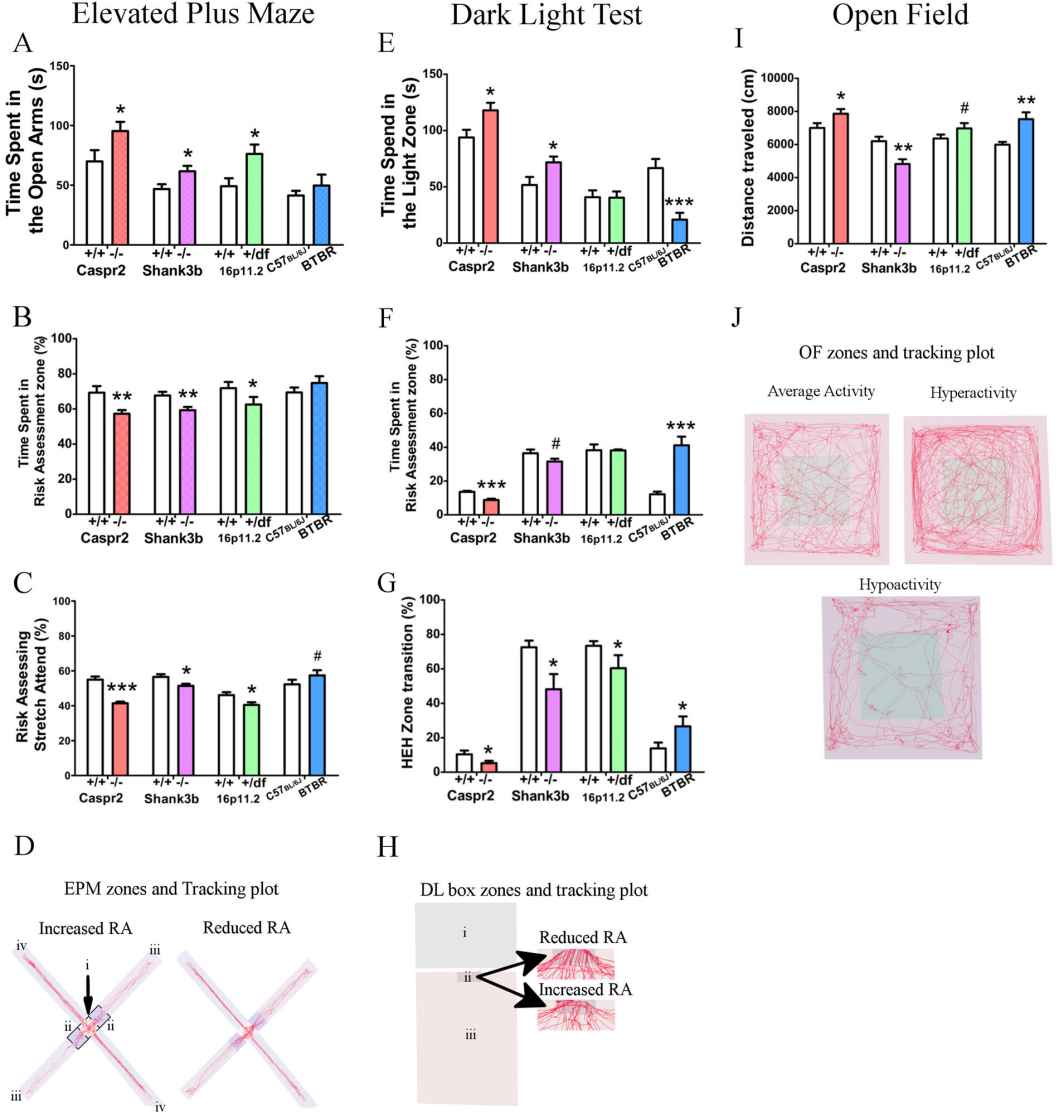
ASD mouse models exhibit anxiolytic and reduced risk-assessing behaviors, and abnormal locomotion. **a** Time spent in the Open Arms for each of the four ASD models and their controls in the EPM. **b** Relative time spent in risk-assessment zone of the four ASD models and their controls in the EPM. **c** Relative risk-assessing stretch-attend of the four ASD mouse models and their controls in the EPM. **d** Representative tracking plots of increased and decreased risk-assessment behaviors, and defined areas used for analysis: (i) the center; (ii) entry zones; (iii) open arms; and (iv) closed arms of the EPM. Risk-assessing was calculated by: (i + 2ii)/(i + 2ii + 2iii). **e** Time spent in the light zone of the four ASD mouse models and their controls in the DL test. **f** Relative time spent in risk-assessment zone of the four ASD models and their controls in the DL test. **g** Relative HEH zone transition of the four ASD mouse models and their controls in the DL test. **h** Representative tracking plot of reduced and increased risk-assessment behavior: (i) dark zone; (ii) entry zone; and (iii) light zone of the DL test. Risk-assessing was calculated thusly: (i/i + ii). **i** Total distance traveled in the OF in four ASD mouse models and their controls. **j** Representative tracking plots for average activity, hyperactivity, and hypoactivity. HEH = Hidden zone → Entry zone → Hidden zone. *CASPR2* KO *n* = 13, WT *n* = 11; *SHANK3b* KO *n* = 10, WT *n* = 10; 16p11.2df *n* = 11, WT *n* = 13; BTBR *n* = 13, C57BL/6J *n* = 13. 0.05 < ^#^*P* < 0.1, *P < 0.05, **P < 0.01, ***P < 0.001. Error bars represent the S.E.M

EPM, DL test, and OF tests were carried out on four separate well-characterized models of ASD: *CASPR2* KO, *SHANK3b* KO (exon 13–16), Chr16p11.2df, and BTBR. In the EPM, the three transgenic models *SHANK3b* KO, *CASPR2* KO, and Chr16p11.2df, spent significantly more time in the open arms relative to their littermate controls, therefore exhibiting anxiolytic behavior. However, there was no difference in the BTBR model (Fig. 1a). In addition, the *CASPR2* KO and *SHANK3b* KO models entered the open arms significantly more while the Chr16p11.2df and BTBR models showed no difference compared to their controls (Supplementary Fig. S2b). Importantly, no changes were observed in entries into the closed arms and in locomotion for *SHANK3b* KO, Chr16p11.2df, and BTBR indicating that their anxiety-like behavior was not influenced by locomotion in the EPM. However, the *CASPR2* KO model showed increased locomotion but no changes in entries into closed arms (Supplementary Fig. S2c, d). In order to understand if the anxiety-like behaviors could be explained by impaired risk-assessment, we studied how much time each mouse spent in a risk-assessment zone (the center and entry zones into the open arms) compared to the entire open area (Fig. 1d). This method has previously been used to examine risk-assessment behavior in the DL test^32^. All three genetic models spent less time in the risk-assessment zone, compared to their controls, while there was no difference in the BTBR model (Fig. 1b). We validated this observation with two different analysis for risk-assessment and exploratory behavior, the stretch-attend behavior and protected head-dips. We measured the relative frequency of the stretch-attend behavior in the risk-assessment zone compared to stretch-attend in the entire EPM. This behavior has previously been shown to reflect risk-assessing behavior^33^. All the three genetic models performed less stretch-attend in the risk-assessment zone, compared to their controls, while there was a tendency for more stretch-attend in the BTBR model (Fig. 1c). In addition, the three transgenic models performed less protected head-dips compared to controls while the BTBR showed increased protected head dips (Supplementary Fig. S2a). These results suggest that decreased risk-assessment may explain the anxiolytic behavior in the EPM. In the DL test, anxiolytic behavior was registered for both the *SHANK3b* KO and *CASPR2* KO models as they spent significantly more time in the light zone while the BTBR spent significantly less time in the light zone (Fig. 1e). Additionally, anxiolytic behavior was observed as significant increase of entries into light zone by the *CASPR2* KO model, while the *SHANK3b* KO and Chr16p11.2df models showed a tendency for increased entries, while the BTBR showed a significant decrease (Supplementary Fig. S3a). In parallel, we determined risk-assessment in the light zone by calculating the amount of time the mouse spent in an entry zone compared to the entire light zone (Fig. 1h). The *CASPR2* KO displayed significantly less risk-assessment and the *SHANK3b* KO mouse displayed a tendency for less risk-assessment, while the BTBR model displayed significantly more risk-assessment. The Chr16p11.2df model displayed no difference compared to its controls (Fig. 1f). In addition, we assessed risk-taking behaviors by measuring the rate at which mice enter the light zone or reentered the hidden zone after entering the entry zone. This was done by comparing the relative frequency the mouse decided to move in the following pattern: hidden zone–entry zone–hidden zone (HEH), which was compared with the total amount of times the mouse decided to move from the hidden zone to the entry zone. The three genetic models performed the HEH zone transition pattern less frequently than the controls, which implies an increase in risk-taking, while the BTBR model showed an increase in HEH frequency (Fig. 1g). Interestingly, changes in light zone velocity was observed for all the four mouse models, where the *CASPR2* KO, Chr16p11.2df, and BTBR showed increased velocity while the *SHANK3b* KO model showed reduced velocity (Supplementary Fig. S3b). In the OF, the BTBR was the only model to show significant anxiogenic behavior as they spent less time in the center, while the Chr16p11.2df model showed anxiolytic behavior (Supplementary Fig. S4a). In addition, we tested for dysregulated locomotion in the OF arena. Similarly to the velocity measurement in the DL test, the BTBR and *CASPR2* KO models showed hyperactivity and the Chr16p11.2df model showed a tendency for hyperactivity, while the *SHANK3b* KO model displayed hypoactivity (Fig. 1i, j). To determine if hypoactivity in the *SHANK3b* KO mice may be due to dysregulation of locomotor function, we performed the rotarod test. The *CASPR2* KO, Chr16p11.2df, and *SHANK3b* KO showed no difference in latency to fall compared to wildtype littermates, while the BTBR mice displayed shorter latency to fall off the rotarod. We believe that the larger size of the BTBR strain compared to C57BL/6J may have contributed to this result. (Supplementary Fig. S4b). Overall, our behavioral findings suggest reduced risk-assessment behavior in multiple ASD mouse models.

### RNA-seq analysis of the dorsal striatum from the four ASD mouse models reveals commonly differentially expressed genes

**Fig. 2:**
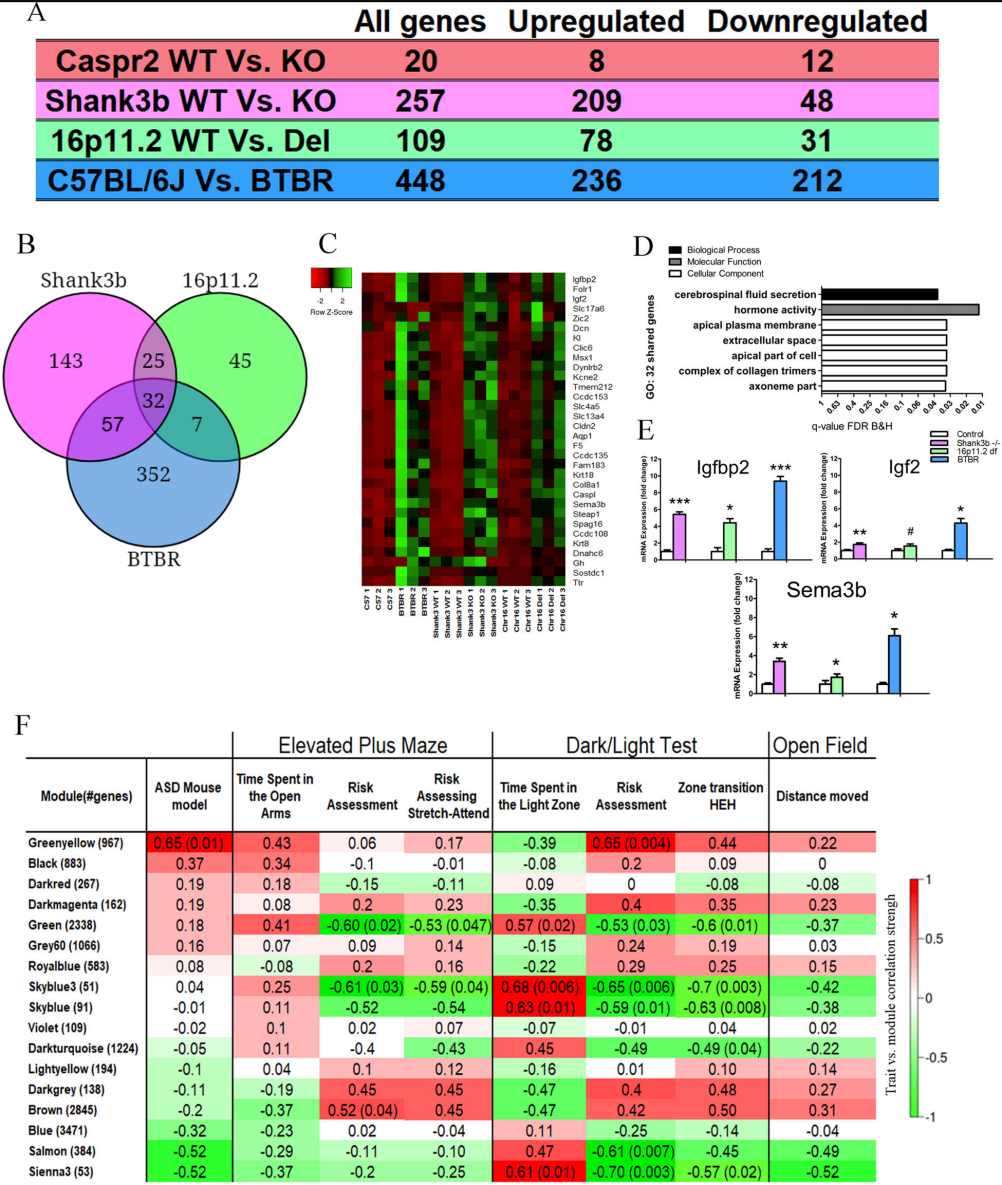
Striatal transcriptome analysis of the four ASD mouse models reveals 32 genes commonly dysregulated in three models and correlations between synchronized gene expression, autism phenotype, and behavioral phenotypes. **a** The number of dysregulated genes in each animal model, including how many were upregulated or downregulated. **b** Venn diagram of commonly dysregulated genes between the *SHANK3b* KO, 16p11.2df, and BTBR models. **c** Heat map of the 32 genes commonly dysregulated in the three animal models. **d** GO analysis of the 32 commonly dysregulated genes. **e** RT-PCR validation in three of the 32 commonly dysregulated genes. **f** Module–trait relationship table. Each cell reports the Pearson correlation value and if significant, the *P*-value in brackets. FDR correction for multiple comparisons was applied on *P*-values. Columns describe the behavioral trait and the rows show the module’s name with number of genes per module in brackets. *n* = 6 for RT-PCR, per genotype per gene. 0.05 < ^#^*P* < 0.1, *P < 0.05, **P < 0.01, ***P < 0.001. Error bars represent the S.E.M

In order to determine specific molecular mechanisms that may be involved in these behaviors, we performed whole genome RNA sequencing on striatal tissue from all mouse models, and their controls (Supplementary Tables S1–S4), followed by a co-expression network analysis to directly correlate gene expression to risk-assessment behaviors. Previous studies have shown that multiple components of the striatum regulate locomotion^34^ and risk-assessment behaviors^4,35^. Additionally, deficits in the striatum have been previously implicated in ASD and several lines of evidence show deficits in striatal medium spiny neurons (MSNs) in both the *SHANK3b* KO and Chr16p11.2df mouse models^24,26^. After sequencing, we performed differential expression analysis and WGCNA^31^, a method used for detecting clusters (modules) of genes which strongly co-express. The module’s eigengenes, which represent the average gene expression in each module, can be linked to different independent traits of the samples^36^. In other words, WGCNA allowed us to directly correlate between the behaviors we observed in our mouse models to mRNA co-expression levels, and thus shed light on possible molecular mechanisms underlying the aberrant behaviors observed. In the differential expression analysis, all four mouse models displayed a variable number of differentially expressed genes, with BTBR showing a high amount of differentially expressed genes (448), and *CASPR2* KO displaying the lowest number of differentially expressed genes (Fig. 2a). Thirty-two genes were commonly dysregulated among the BTBR, *SHANK3b* KO, and Chr16p11.2df mouse models, while no shared genes were found from the *CASPR2* KO model (Fig. 2b, c). We performed gene ontology (GO) analysis on the 32 commonly dysregulated genes and the significant differentially expressed genes from each ASD mouse model (Fig. 2d and Supplementary Fig. S5a–d). Interestingly, we found that the molecular mechanism “hormone activity” was enriched in the 32 commonly dysregulated genes, as well as for the *SHANK3b* KO and Chr16p11.2df models individually. To validate the RNAseq results we performed real-time PCR on the *SEMA3B*, *IGF2*, and *IGFBP2* genes, which were commonly upregulated in the three mouse models (Fig. 2e).

### Weighted gene co-expression network analysis reveals molecular mechanisms which correlate with risk-assessment and anxiolytic behaviors

To discover networks of co-expressed genes that correlate with the behavioral phenotypes, particularly the risk-assessment behaviors, we performed WGCNA on the striatal transcriptome data (Supplementary Table 5). WGCNA clustering resulted in 18 modules of co-expressed genes. The Greenyellow module, which consists of 966 genes displayed a significant positive correlation with the mouse model’s type (ASD mouse model) (Fig. 2f). In other words, these genes were correlated with autism genotype. Correlation analysis between modules and behavioral phenotypes revealed that the Green module (2338 genes) was negatively correlated to risk-assessing, stretch-attend and protected head dips, and negatively correlated to risk-assessing behavior in the DL test and HEH zone transition. In addition, the Green module was positively correlated to time spent in the light zone and entries into light zone in the DL test (Fig. 2f and Supplementary Fig. S6). To improve our understanding of the possible molecular mechanisms these gene modules regulate, we performed GO and PPI analysis on these modules. The Greenyellow module was enriched for developmental-related terms, such as “anatomical structure formation involved in morphogenesis” (Supplementary Fig. 6B). PPI analysis of the Greenyellow module resulted in several clusters. The largest cluster consisted of 46 highly interacting proteins, with several central hubs, such as the Aurka and Aurkb kinases which are involved in chromosomal segregation during meiosis and mitosis (Supplementary Fig. 6c). GO analysis of this cluster enriched for terms related to cell division, such as “cell cycle”, “nuclear division” and “spindle” (Supplementary Fig. S6d).

### Network analysis of the Green module reveals a potential role for abnormal glutamate signaling in atypical risk-assessment and anxiolytic behaviors

**Fig. 3:**
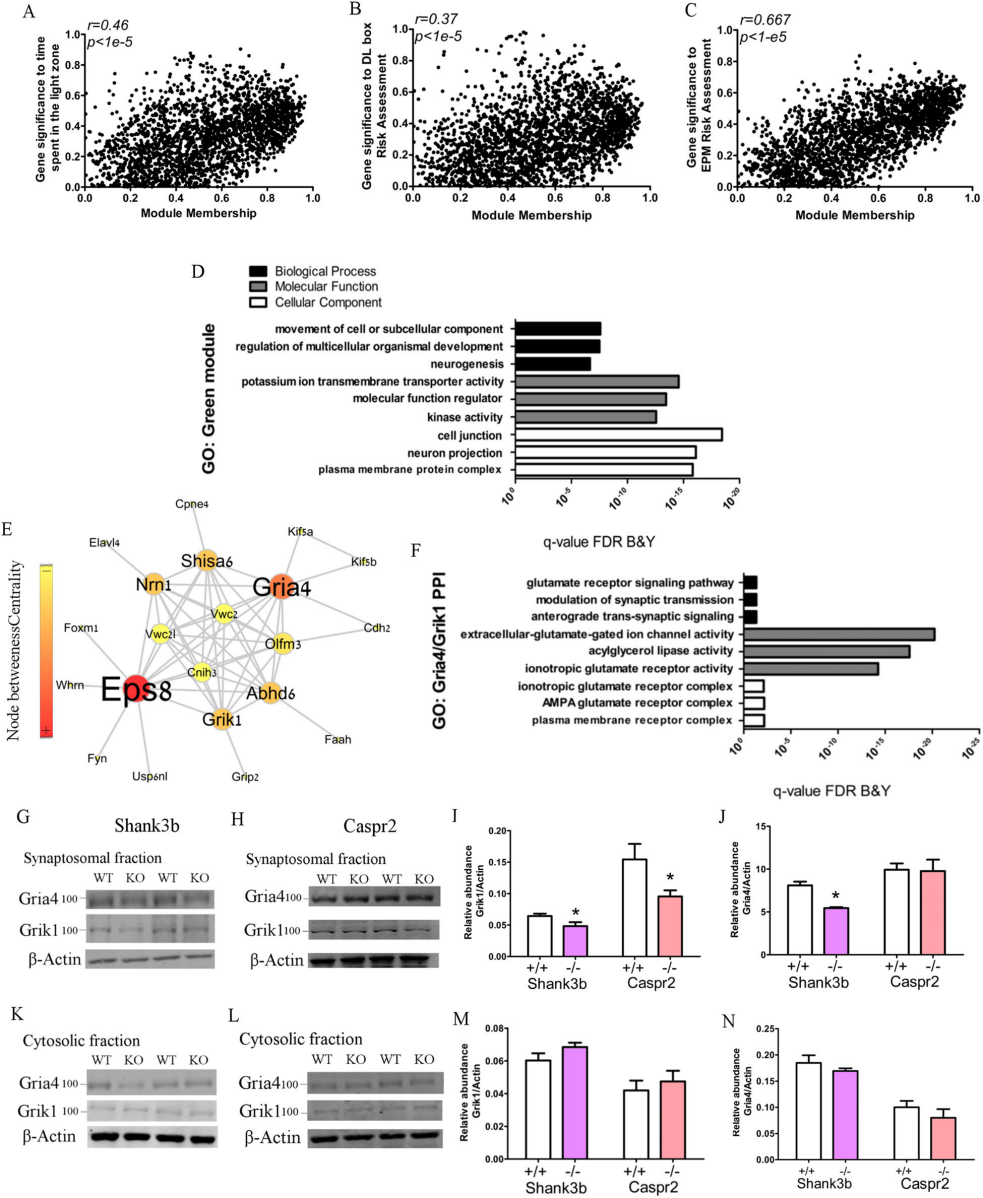
In-depth analysis of the Green module reveals a glutamate signaling PPI network. **a–c** Scatter plots indicate the correlation (Pearson’s *r* correlation coefficient) and its significance (*P*-value) between gene significance (correlation between gene expression and behavior) (*y*-axis) and Module Membership (*x*-axis) for the Green module. **d** Gene ontology enrichment for the Green module. **e** Gria4/Grik1 PPI hub network derived from the Green module. Node size indicate degree; the number of interactors. Color indicates BetweenessCentrality of node within the network. **f** GO analysis of the Gria4/Grik1 PPI. **g** Western blot of Gria4 and Grik1 in the synaptosomal fraction of *SHANK3b* dorsal striatum. **h** Western blot of Gria4 and Grik1 in the synaptosomal fraction of *CASPR2* dorsal striatum. **i** Quantification of Grik1 in the synaptosomal fraction of *SHANK3b* and *CASPR2* animal models. **j** Quantification of Gria4 in the synaptosomal fraction of the *SHANK3b* and *CASPR2* animal models. **k** Western blot of Gria4 and Grik1 in the cytosolic fraction of *SHANK3b* dorsal striatum. **l** Western blot of Gria4 and Grik1 in the cytosolic fraction of *CASPR2* dorsal striatum. **m** Quantification of Grik1 in the cytosolic fraction of *SHANK3b* and *CASPR2* animal models. **n** Quantification of Gria4 in the cytosolic fraction of the *SHANK3b* and *CASPR2* animal models. *n* = 6 for WB, per genotype per gene. *P < 0.05. Error bars represent the S.E.M

Considering that the Green module had strong correlations with risk-assessment behavior (Fig. 2f), we further examined the genes found in this module. First, we verified that the strength of membership of a gene to the Green module is positively correlated to the gene’s correlation to the risk-assessment behaviors (Fig. 3a–c). This gives further evidence of the association of these genes to risk-assessment behaviors. GO analysis of the top coexpressed genes in the Green module revealed enrichment for the terms “neurogenesis”, “kinase activity”, and “neuron projections” (Fig. 3d). PPI analysis of the Green module revealed one cluster that included 22 genes (Fig. 3e), with 11 being tightly interconnected, which included two hubs of particular interest: the glutamate AMPA receptor subunit Gria4 and the kainate receptor subunit Grik1. In addition, two proteins involved in AMPA receptor trafficking and channel gating, Grip2 and Cnih3 were also part of the network. GO analysis of this cluster enriched for glutamate neurotransmission-related terms such as “glutamate receptor singling pathway” and “extracellular-glutamate-gated ion channel activity” (Fig. 3f). These results suggest that dysregulation of glutamate signaling may be involved in the risk-assessment behavior of ASD mice models. Additional modules (e.g. Skyblue, Skyblue3, and Sienna3) also showed correlations with anxiety-like and risk-assessing behaviors, however the number of genes which composed these modules was relatively small (Fig. 2f). The subsequent PPI analysis did not yield strongly interconnected networks (Supplementary Fig. S7a–c), and no GO terms were enriched as well. Therefore, we further focused on the dysregulation of the striatal glutamatergic network in the Green module.

Because mRNA levels do not necessarily predict functional protein levels^37^, we further determined if glutamate receptor subunit levels are dysregulated in the dorsal striatum of *SHANK3b* and *CASPR2* animals, by performing western blot on Gria4 and Grik1 in synaptosomal and cytosolic fractions from the dorsal striatum. We chose the *SHANK3b* model due to the previous reports of striatal dysregulation in this mouse model^24^, while the *CASPR2* KO model showed the most consistent dysregulation of anxiety and risk-assessment in our initial behavioral experiments (Fig. 1). The western blot analysis showed that Grik1 significantly reduced in the synaptosomal fraction of *SHANK3b* KO and *CASPR2* KO mice (Fig. 3g–i), while there were no differences in the cytosolic fractions (Fig. 3k–m). The levels of Gria4 showed significant reduction in the synaptosomal fraction of *SHANK3b* KO, while no changes were observed in the *CASPR2* animals (Fig. 3g, h, j). No changes were observed for the Gria4 in the cytosolic fraction for both animals (Fig. 3k, l, n). This result verifies the dysregulation of the glutamatergic system in the dorsal striatum but suggests a more complex interaction between transcriptome and protein changes. Therefore, to extend our understanding of the functional significance of glutamatergic signaling in risk-assessment behavior, we performed pharmacological studies.

### Pharmaceutical inhibition of AMPA and Kainate receptors recapitulates risk-assessment and anxiolytic behaviors in C57BL/6J mice

**Fig. 4:**
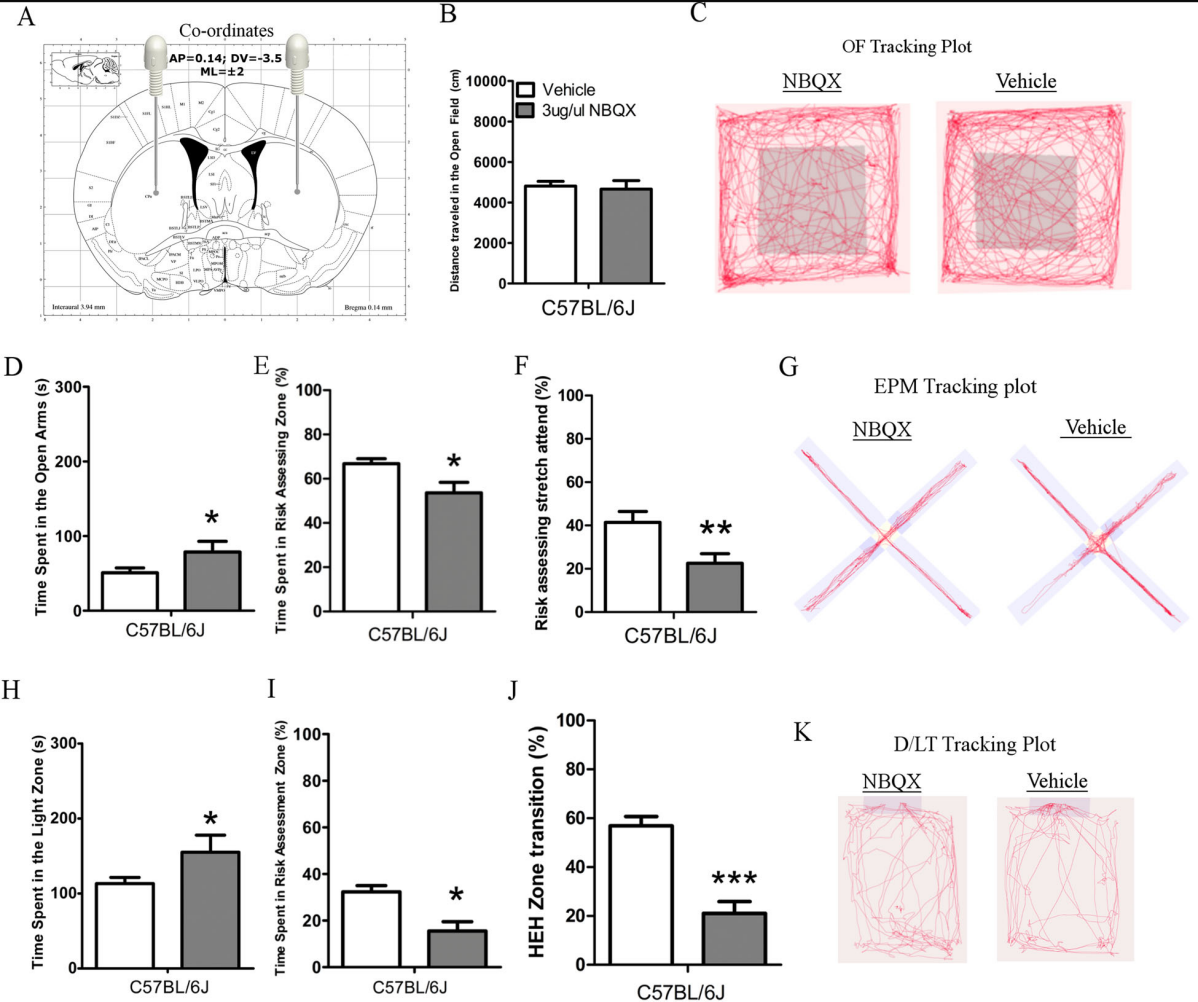
Pharmacological inhibition of striatal glutaminergic receptors recapitulates reduced risk-assessment and reduced anxiety-like behavior in C57BL/6J mice. **a** Illustration of Cannula insertion co-ordinates into the mouse dorsal striatum. The mouse brain illustration is taken from The Mouse Brain in Stereotaxic Coordinates, Compact, Third Edition^47^. CPu caudate putamen; AP anterior–posterior; DV dorsal ventral; ML medial lateral. **b** Total distance traveled by C57BL/6J mice after NBQX or vehicle treatment in the OF. **c** Representative tracking plot of the OF. **d** Time spent in the open arms by the C57BL/6J after NBQX or vehicle treatment in the EPM. **e** Relative time spent in risk-assessment zone by C57BL/6J after NBQX or vehicle treatment in the EPM. **f** Relative stretch attend postures performed by C57BL/6J mice after NBQX or vehicle treatment in the EPM. **g** Representative tracking plots in the EPM. **h** Time spent in the light zone C57BL/6J after NBQX and vehicle treatment in the DL test. **i** Relative time spent in risk-assessment zone C57BL/6J after NBQX and vehicle treatment in the DL test. **j** Relative HEH zone transition by C57BL/6J after NBQX and vehicle treatment in the DL test. **k** Representative tracking plots of the DL. C57BL/6J NBQX *n* = 11, Vehicle *n* = 10. *P < 0.05, **P < 0.01, ***P < 0.001. Error bars represent the S.E.M

To determine if dysregulation of striatal AMPA and Kainate receptor signaling may play a functional role in the risk-assessment behaviors seen in autism models, we performed administration of an AMPA/Kainate inhibitor, 2,3-dihydroxy-6-nitro-7-sulfamoyl-benzo[f]quinoxaline-2,3-dione (NBQX) into the dorsal striatum (Fig. 4a) of C57BL/6J mice, followed by behavioral testing. NBQX had no effects on locomotion in the OF and the EPM, as well as no changes in velocity in the DL test (Fig. 4b, c, Supplementary Fig. S8a, e). In the EPM, NBQX infusion induced an increase in time spent in the open arms, increased entries into the open arms and decrease in protected head-dips (Fig. 4d, g, Supplementary Fig. S8b, c), as well as significant decrease in risk-assessment time (Fig. 4e), and relative stretch-attend (Fig. 4f). In the DL test, NBQX infusion increased time spent in the light zone (Fig. 4h, k) and reduced risk-assessment and HEH transition (Fig. 4i, j). Therefore, inhibition of striatal AMPA signaling in C57BL/6J mice mimics the decreased risk-assessment behavior seen in multiple ASD models and has no general effects on locomotion. These results are in line with the lack of significant correlation between the Green module and the “distance moved” trait in the OF, EPM, and velocity in the DL test, and therefore seems to not be involved with the regulation of this behavior (Fig. 2f and Supplementary Fig. S6).

### Pharmaceutical inhibition of AMPA and Kainate receptors exacerbates risk-assessment and anxiolytic behaviors in the *CASPR2 KO* and *SHANK3b* KO mice

**Fig. 5:**
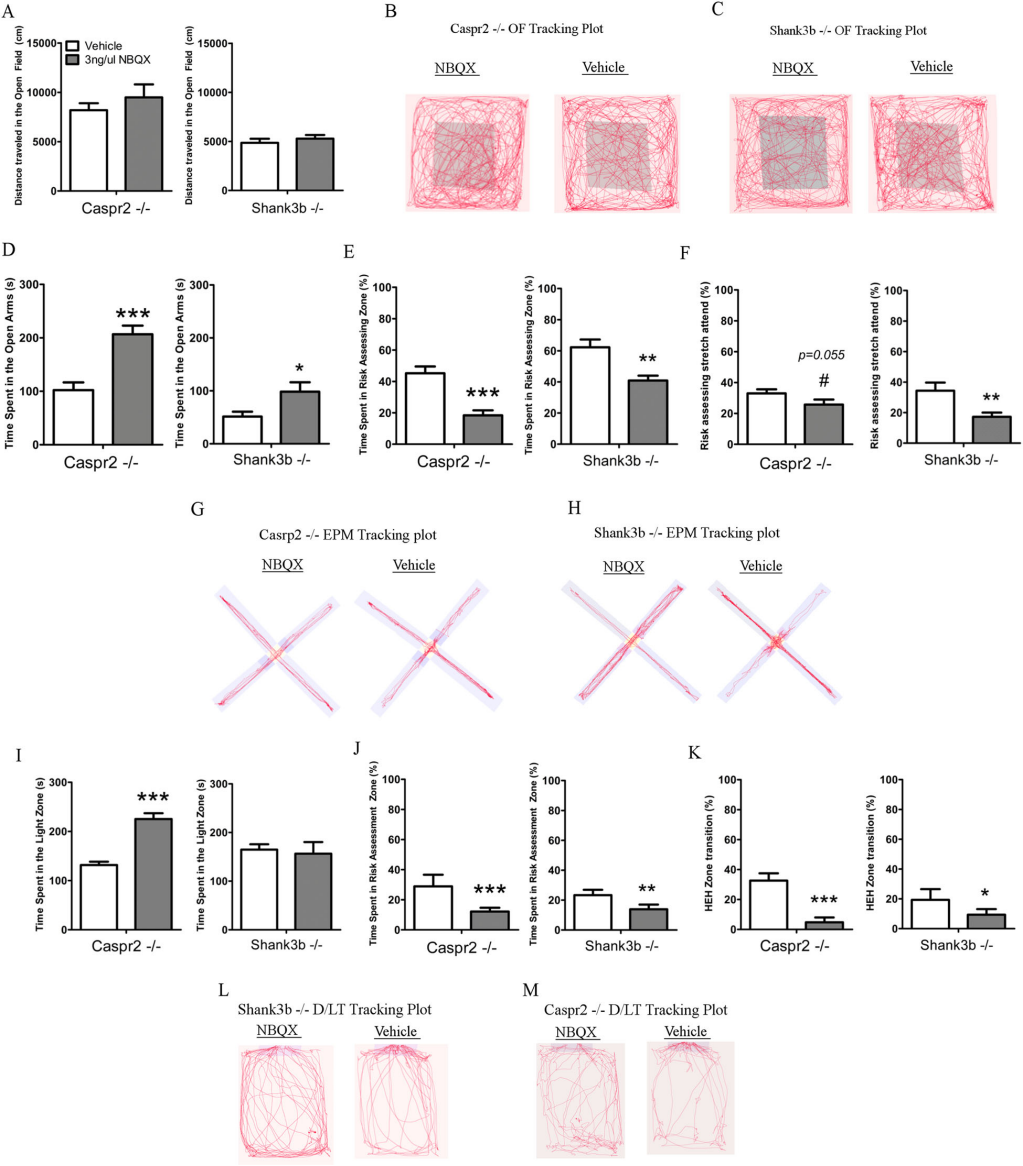
Pharmacological inhibition of striatal glutaminergic receptors exacerbates risk-assessment and anxiolytic behaviors in *SHANK3b* KO and *CASPR2* KO without influencing locomotion. **a** Total distance traveled by *CASPR2* KO and *SHANK3b* KO mice after NBQX or vehicle treatment in the OF. **b** Representative tracking plots of *CASPR2* KO in the OF test. **c** Representative tracking plots of *SHANK3b* KO in the OF test. **d** Time spent in the open arms by the *CASPR2* KO and *SHANK3b* KO after NBQX and vehicle treatment in the EPM. **e** Relative time spent in risk-assessment zone by *CASPR2* KO and *SHANK3b* KO mice after NBQX or vehicle treatment in the EPM. **f** Relative stretch attend postures performed by *CASPR2* KO and *SHANK3b* KO mice after NBQX or vehicle treatment in the EPM. **g** Representative tracking plots of *CASPR2* KO in the EPM. **h** Representative tracking plots of *SHANK3b* KO in the EPM. **i** Time spent in the light zone by *CASPR2* KO and *SHANK3b* KO after NBQX and vehicle treatment in the DL test. **j** Relative time spent in risk-assessment zone by *CASPR2* KO and *SHANK3b* KO after NBQX and vehicle treatment in the DL test. **k** Relative HEH zone transition by *CASPR2* KO and *SHANK3b* KO after NBQX and vehicle treatment in the DL test. **l** Representative tracking plots of *CASPR2* KO in the DL test. **m** Representative tracking plots of *SHANK3b* KO in the DL test. HEH = Hidden zone → Entry zone → Hidden zone. *SHANK3b* KO NBQX *n* = 9, Vehicle *n* = 10. *CASPR2* KO NBQX *n* = 10–9, Vehicle *n* = 10. 0.05 < ^#^*P* < 0.1, *P < 0.05, **P < 0.01, ***P < 0.001. Error bars represent the S.E.M

To better understand if reduced AMPA/Kainite signaling is involved in reduced risk-assessment, we inhibited AMPA/Kainate signaling in *CASPR2* KO and *SHANK3b* KO models. This was performed to determine if NBQX can also have these effects on mice with ASD-associated genetic variation. Similar to the C57BL/6J mice infused with NBQX, both the *CASPR2* KO and *SHANK3b* KO models showed no changes in distance traveled in the OF, EPM, and velocity in the DL test (Fig. 5a–c, Supplementary Fig. S9a, d). In the EPM, NBQX infusion increased time spent in the open arms, increased entries into open arms and reduced protected head dips for *CASPR2* KO and *SHANK3b* KO animals (Fig. 5d, g, h, and Supplementary Fig. S9b, c). Exacerbation was also observed in risk-assessment as NBQX infusion decreased time spent in risk-assessment zone for both models, and decreased stretch-attend postures were observed for *SHANK3b* KO while a tendency for reduction (*P* = 0.055) was observed for *CASPR2* KO (Fig. 5e, f). In the DL test, NBQX infusion increased time spent in the light zone for the *CASPR2* KO, while no change was observed in the *SHANK3b* KO model, and there were no changes in entries into the light zone (Fig. 5i, l, m and Supplementary Fig. S9e). Additionally, NBQX infusion reduced time spent in risk-assessment zone and HEH transition for both models (Fig. 5j, k). Therefore, our pharmacological studies reveal that inhibition of striatal glutaminergic signaling can modulate risk-assessment behaviors in mice.

## Discussion

By comparing multiple male ASD mouse models, we observed a dysregulation in risk-assessment behaviors. By characterizing the striatal transcriptome of these animal models, using WGCNA and PPI network analysis, and subsequently performing pharmaceutical in vivo validation, we found a glutaminergic signaling pathway which regulates these behaviors.

In the previous studies examining comorbid-like behaviors in the genetic models, we focused mainly on anxiety-like behaviors and locomotion^25,26,38,39^. In our initial behavioral experiments, the transgenic models expressed anxiolytic behaviors, differences in locomotion, and deficient risk-assessment behavior. While the anxiolytic behavior was an initial surprise (as is discussed later), the anxiety-like behavior correlated with risk-assessment in all tested animal models. Risk-assessment behavior has not previously been characterized in ASD mouse models.

Regarding anxiety-like behaviors, it may be expected that ASD mouse models would express anxiogenic behavior, considering reports of increased anxiety in humans diagnosed with ASD. However, previous mouse behavioral studies are inconclusive regarding the anxiety-like phenotype. For example, in a study where the homer domain of *SHANK3* was abolished (exon 21), the KO mice showed anxiogenic behavior in the DL test but not in the EPM and OF^40^, while in studies where the ankyrin domain was abolished (exons 4–9), there was no observed anxiety-like behavior when tested in the EPM and DL tests^41,42^. Previous studies experimenting with the Shank3 model we use in our study (*SHANK3b)*, where the SH3 domain is abolished (exon 13–16), have reported varied anxiety-like behaviors. One study showed that the *SHANK3b* KO mice exhibited anxiogenic behavior in the elevated zero maze (EZM)^24^. However, in a different study testing two cohorts of *SHANK3b*, there was no difference in the EPM in both cohorts^38^. The differences between the anxiolytic phenotype we observed in the EPM and the anxiogenic behaviors previously shown in the EZM could be attributed to the center domain of the EPM, as it might influence the mouse decision-making process, and therefore influence the mouse’s reaction to the maze environment. Supporting this assumption are studies focused on BTBR, which showed that in the EZM, BTBR mice spend significantly more time in the open arms^39^, while in the EPM they spend less time in the open arms^43^, suggesting that the EZM and the EPM elicit different anxiety-like behaviors.

We observed changes in risk-assessment and risk-taking behaviors that correlated with the anxiety-like behaviors across the four animal models. Previous studies examining risk-assessing and risk-taking behaviors in adolescents diagnosed with ASD using the analog balloon test have shown a positive relationship between anxiety and risk-taking behavior^5,6^. This relationship may represent a unique manifestation of anxiety in people diagnosed with ASD. This may also explain the increased time spent in open areas in the anxiety tests among our ASD transgenic models. Anxiety associated with ASD may manifest itself differently compared to classic anxiety.

An important limitation of mouse behavioral studies in general, and the mazes used in our study in particular, is that results may be cofounded by dysregulation of locomotion. This is also important considering that our mouse models display differences in locomotion (although in most cases, this cannot be characterized by dysregulation, but more as hyperlocomotion). To address this issue we measured the relative frequency of the ethological stretch-attend behavior performed by the animals in the regions dedicated to risk-assessment in the EPM, which has previously been shown to be independent of locomotion^33^, as well as measured risk-taking behavior in the DL test (HEH). In addition, we analyzed protected head-dips, a type of exploratory/risk-assessing behavior, which is unrelated to locomotion. In all these analysis of risk-assessment, the transgenic mouse models showed significant reduction while the BTBR showed a significant increase.

To associate specific molecular pathways to the behaviors we observed, we performed WGCNA on striatal transcriptome data, which allowed us to detect specific groups of genes which coexpress in the dorsal striatum of the mouse models. We chose the dorsal striatum, as this brain region has previously shown to be central to risk-driven decision-making in human studies and in risk-assessment and risk-taking behavior in animal models^8,9^. Multiple modules of coexpressed genes showed a significant correlation with risk-assessment behaviors. In contrast, no module correlated significantly with measurements of locomotion, which implies that risk-assessment and locomotion are regulated by different molecular mechanisms in the dorsal striatum. The Green module negatively correlated to risk-assessment behavior and positively correlated with anxiolytic behavior. In-depth analysis of the green module using PPI analysis revealed a subnetwork that included the Gria4 and Grik1 ionotropic subunits. Interestingly, at the expression level, Gria4 and Grik1 increased in multiple models including *SHANK3b* KO and *CASPR2* KO, however at the protein level we found a decrease in Gria4 and Grik1 levels in the synapses of *SHANK3b* KO and a decrease in Grik1 levels in the synapses of *CASPR2* KO. The Shank3 PDZ domain—which is abolished in *SHANK3b* KO—binds AMPA receptors to the post synaptic membrane^44^, and reduced AMPA-mediated mEPSCs were previously observed in the dorsal striatum of *SHANK3b* KO mice^24^. Therefore, the reduced synaptic levels of Gria4 and Grik1 we observed in *SHANK3b* KO might contribute to mEPSC dysregulation. This data may explain the contrast we observed between the expression and the protein levels of Gria4 and Grik1^45^. Put differently, the reduced levels of Gria4 and Grik1 in the synapse dysregulates neuronal excitability, which may initiate downstream signaling that increases gene expression as a compensation mechanism^46^.

The reduced levels of Gria4 and Grik1 in the synaptosomal fraction of the dorsal striatum of the *SHANK3b* KO and the reduction of Grik1 in the *CASPR2* KO mice led to the hypothesis that inhibition of these subunits by NBQX infusion will recapitulate the reduced risk-assessment and anxiolytic behaviors in C57BL/6J mice, which we were able to verify in vivo. We were also able to exacerbate these behaviors in *SHANK3b* KO and *CASPR2* KO by the same approach, which strengthened the involvement of AMPA/Kainate neurotransmission in the dorsal striatum to risk-assessment and anxiolytic behavior. Of importance, NBQX had no effects on locomotor activity in any of our experiments. This strengthens the claim that glutaminergic signaling in the dorsal striatum is specifically involved in risk-assessment behavior, and not in the locomotion dysfunction seen in autism mouse models.

To conclude, these results are, to the best of our knowledge, the first to indicate a common biological mechanism that regulates risk-assessment behaviors in ASD mouse models. In addition, by validating the PPI network analysis by performing in vivo pharmaceutical experiments, we demonstrate that this approach has a potential in discovering additional targets that underlay the manifestation of specific phenotypes associated with ASD.

## Supplementary information

Supplementary Figures

Supplementary Table 1

Supplementary Table 2

Supplementary Table 3

Supplementary Table 4

Supplementary Table 5

Supplementary Table 6
