# Supplementary material for: Gene network analysis reveals a role for striatal glutamatergic receptors in dysregulated risk-assessment behavior of autism mouse models
Source: Transl Psychiatry. 2019 Oct 17;9:257. doi: 10.1038/s41398-019-0584-5 (PMC6797764; doi:10.1038/s41398-019-0584-5)
Supplement: Supplementary file 1 — Supplementary Figures [file 41398_2019_584_MOESM1_ESM.docx]

Gene network analysis reveals a role for striatal glutamatergic receptors in dysregulated risk-assessment behavior of autism mouse models

Supplemental Figures and Tables


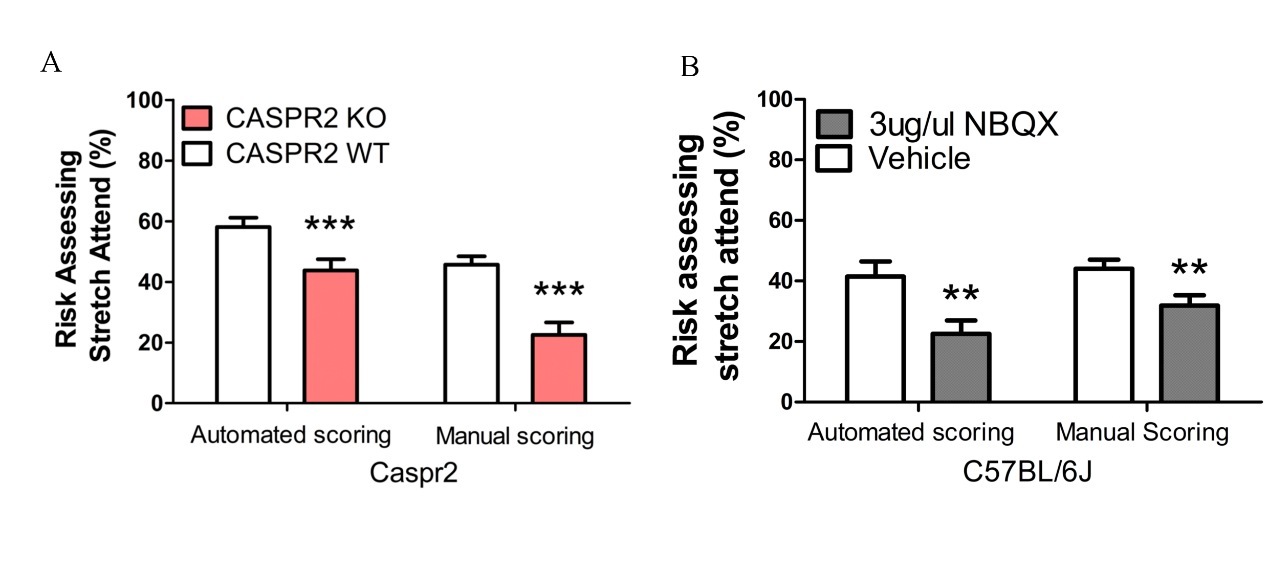


**Supplementary Figure S1 | Manual scoring Vs. automated scoring of stretch-attend postures** (A) Scoring from the initial *CASPR2* behavioral experiments. (B) Scoring from the C57BL/6J NBQX infusion experiment. *CASPR2* KO n=13, WT n=11. C57BL/6J NBQX n=11, Vehicle n=10. 0.05<#P<0.1, *P<0.05, **P<0.01, ***P<0.001. Error bars represent the S.E.M.

**
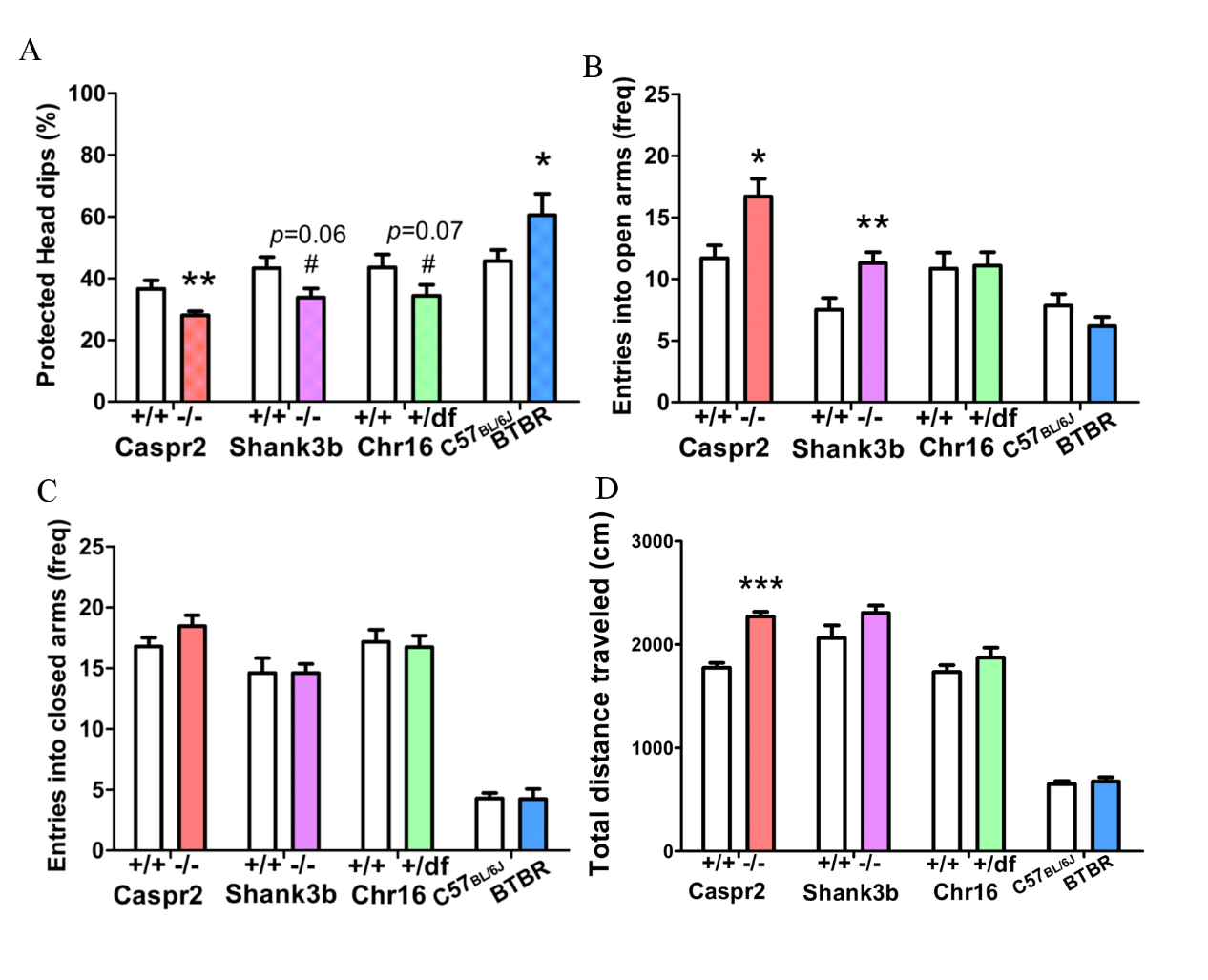
Supplementary Figure S2** | **Locomotion and additional anxiety-like behaviors in the EPM for the four ASD mouse models** (A) Relative frequency of head dips in the risk-assessment zone of the EPM performed by the four ASD models and their relative controls. (B) Entries into the open arms of the EPM for each of the four ASD mouse models and their controls. (C) Entries into the closed arms of the EPM for each of the four ASD mouse models and their control. (D) Total distance traveled in the EPM for each of the four ASD mouse models and their controls. *CASPR2* KO n=13, WT n=11; *SHANK3b* KO n=10, WT n=10; 16p11.2df n=11, WT n=13; BTBR n=13, C57BL/6J n=13. 0.05<#P<0.1, *P<0.05, **P<0.01, ***P<0.001. Error bars represent the S.E.M.


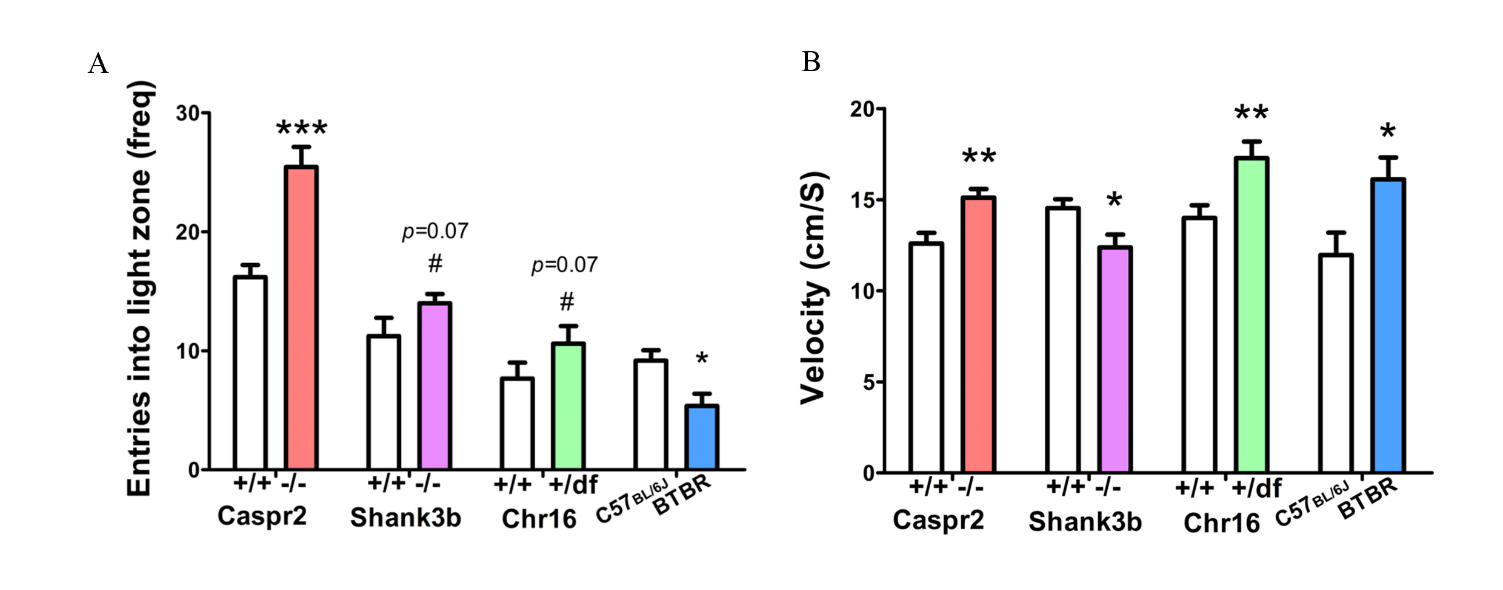


**Supplementary Figure S3 | Locomotion and additional anxiety-like behaviors in the DL test for the four ASD mouse models** (A) Entries into the light zone of the DL for each of the four ASD mouse models and their controls. (B) Velocity in the light zone of the DL for the four ASD mouse models and their controls. *CASPR2* KO n=13, WT n=11; *SHANK3b* KO n=10, WT n=10; 16p11.2df n=11, WT n=13; BTBR n=13, C57BL/6J n=13. 0.05<#P<0.1, *P<0.05, **P<0.01, ***P<0.001. Error bars represent the S.E.M.

**
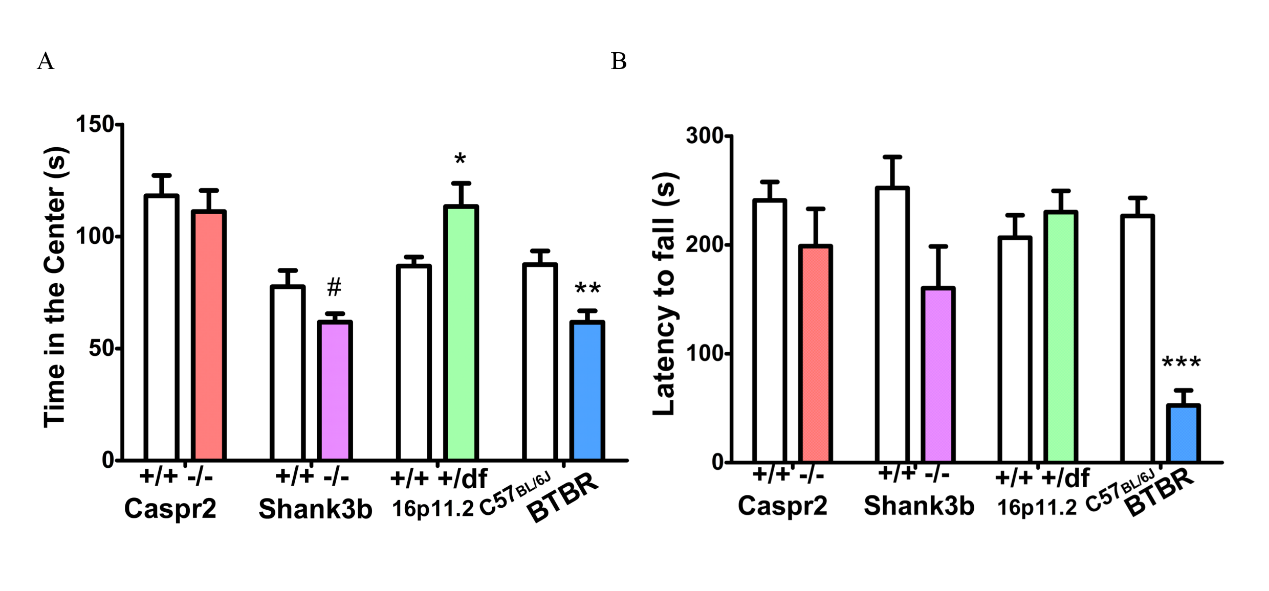
**

**Supplementary Figure S4 | Rotarod and time spent in the center of the OF test for the four ASD mouse models** (A) Time spent in the center of the OF for the four ASD mouse models and their controls. (B) Latency to fall from the rotarod first for the four ASD mouse models and their controls. *CASPR2* KO n=13, WT n=11; *SHANK3b* KO n=10, WT n=10; 16p11.2df n=11, WT n=13; BTBR n=13, C57BL/6J n=13. 0.05<#P<0.1, *P<0.05, **P<0.01, ***P<0.001. Error bars represent the S.E.M.

**
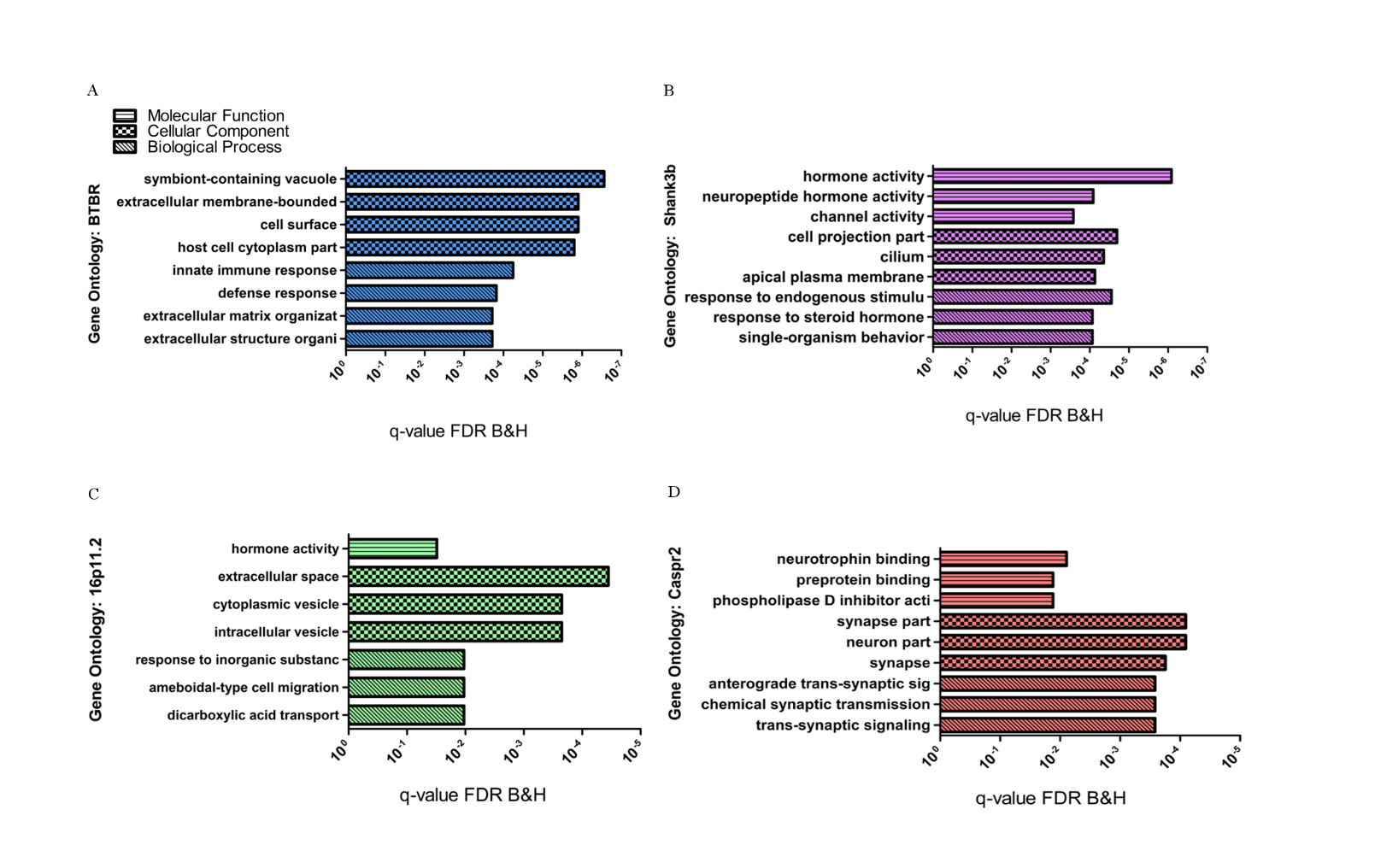
Supplementary Figure S5 | Gene Ontology enrichment for the differentially expressed genes in the dorsal striatum of each ASD animal model and Aurka subppi.** (A) Gene Ontology enrichment of the differentially expressed genes for the BTBR model. (B) Gene Ontology enrichment of the differentially expressed genes for the *SHANK3b* KO model. (C) Gene Ontology enrichment of the differentially expressed genes for the Chr16p11.2df. (D) Gene Ontology enrichment of the differentially expressed genes for the *CASPR2* KO model.
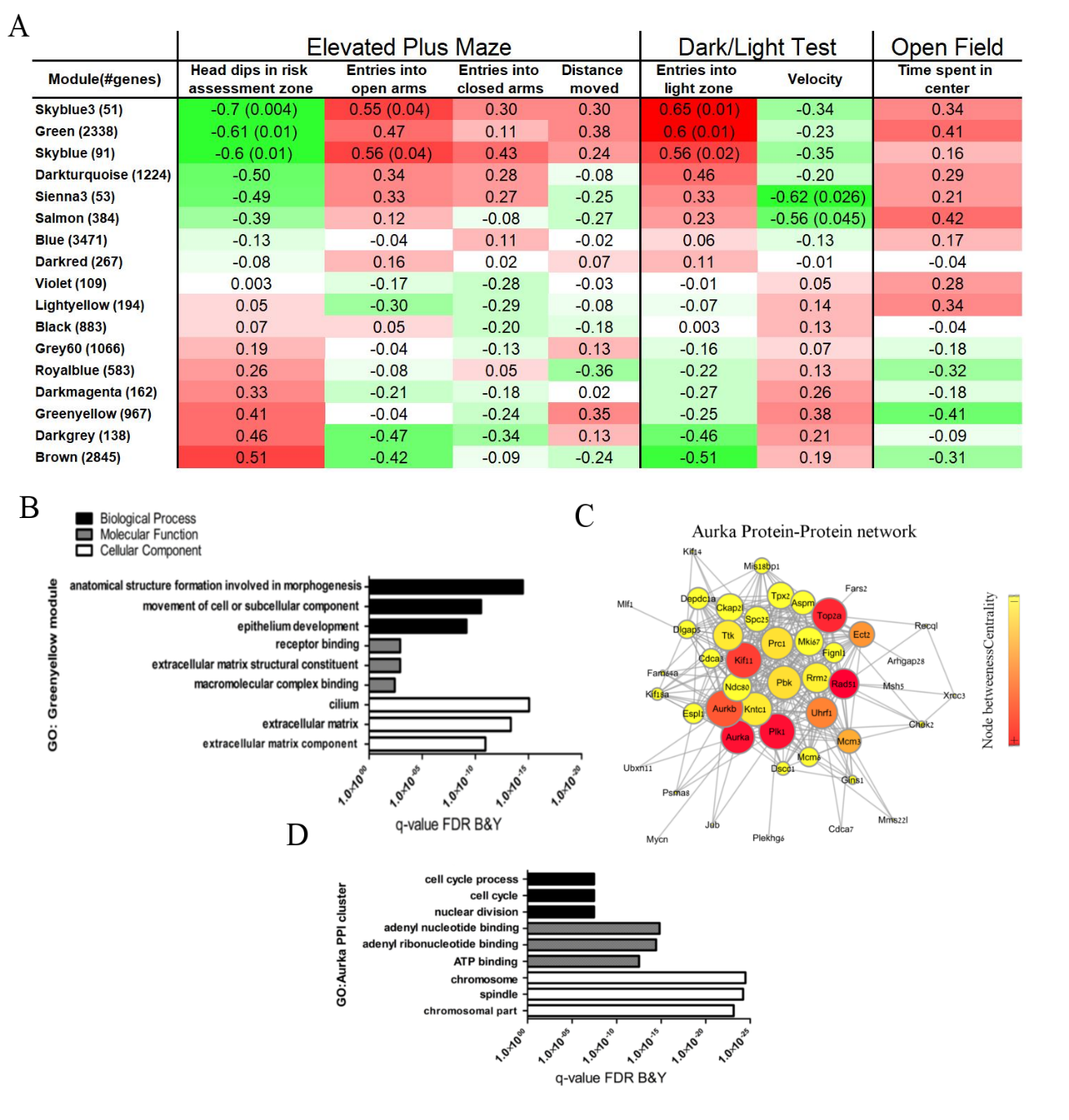


**Supplementary Figure S6 | Module-trait relationship table for the additional behaviors measured**. (A) Each cell reports the Pearson correlation value and if significant, the *p*-value in brackets. FDR correction for multiple comparisons was applied on *p*-values. Columns describe the behavioral trait and the rows show the module's name with number of genes per module in brackets. (B) GO analysis for the Greenyellow module. (C) The Aurka PPI network. Node size reflects number of interactions with other nodes. Node color reflects BetweenessCentrality value within. (D) Gene Ontology enrichment of the Greenyellow Aurka subppi.


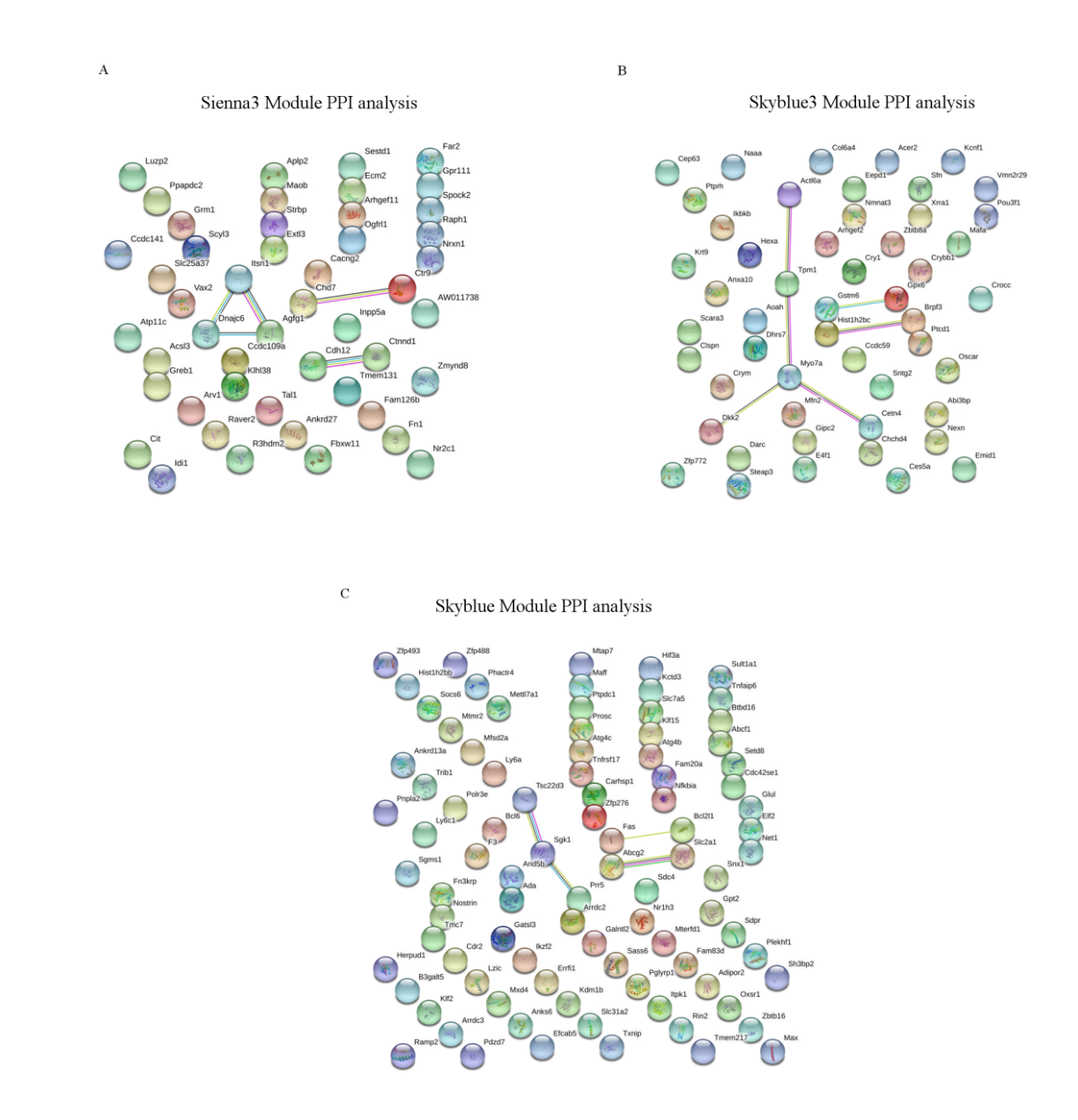


**Supplementary Figure S7 |** **Protein-Protein Interaction (PPI) analysis of Skyblue, Skyblue3 and Sienna3 modules show little interconnectivity.** (A) PPI network enrichment for the Skyblue module from the STRING database. (B) PPI network enrichment for the Skyblue3 module from the STRING database. (C) PPI network enrichment for the Sienna3 module from the STRING database.


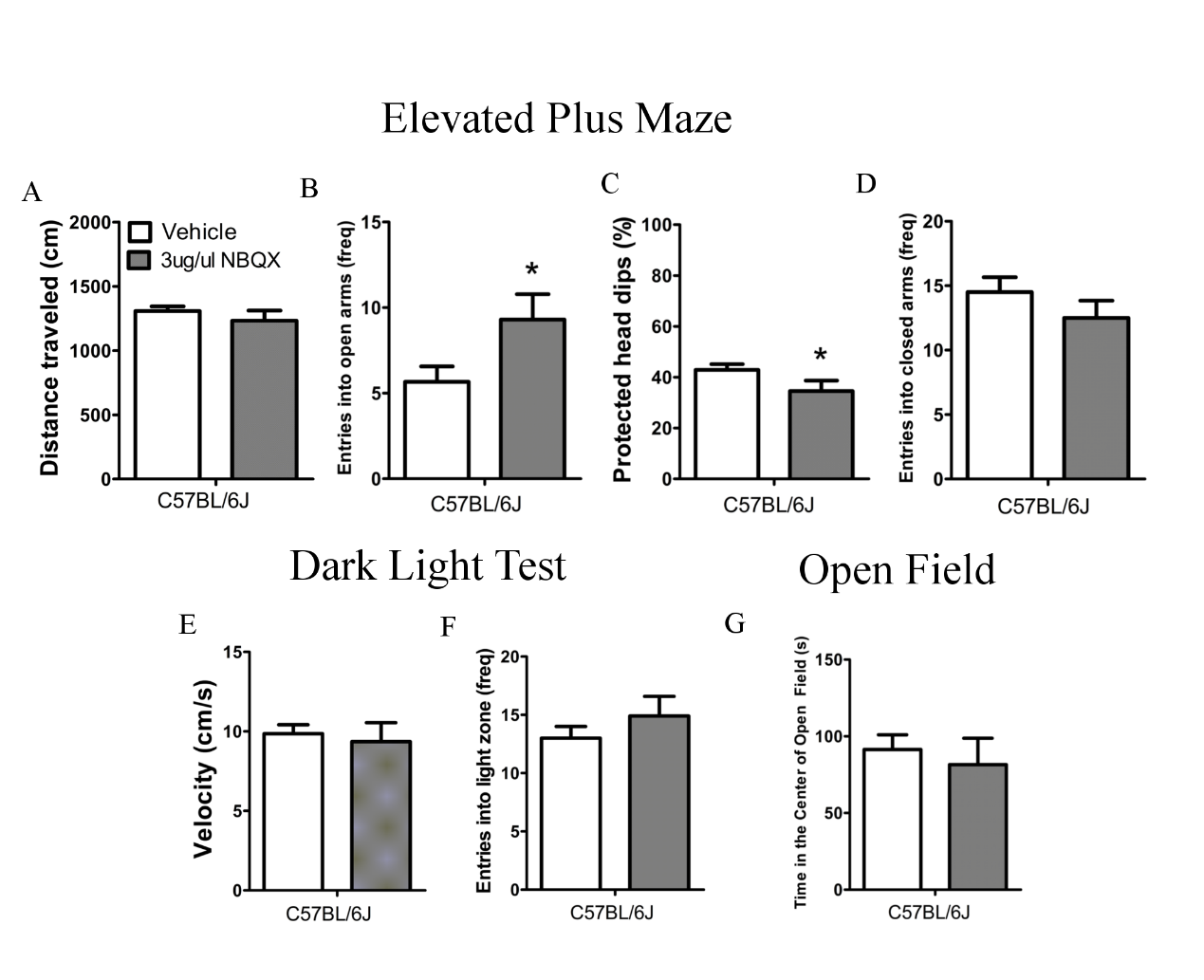
**Supplementary Figure S8 | Locomotion and additional anxiety-like behaviors in the C57BL/6J glutamate inhibition experiment.** (A) Total distance traveled in the EPM for C57BL/6J mice after NBQX or vehicle treatment. (B) Entries into the open arms of the EPM for C57BL/6J mice after NBQX or vehicle treatment. (C) Relative frequency of head dips in the risk-assessment zone of the EPM performed by C57BL/6J mice after NBQX or vehicle treatment. (D) Entries into the closed arms of the EPM for C57BL/6J mice after NBQX or vehicle treatment. (E) Velocity in the light zone of the DL for C57BL/6J mice after NBQX or vehicle treatment. (F) Entries into the light zone of the DL for C57BL/6J mice after NBQX or vehicle treatment. (G) Time spent in the center of the OF for C57BL/6J mice after NBQX or vehicle treatment. C57BL/6J NBQX n=11, Vehicle n=10. 0.05<#P<0.1, *P<0.05, **P<0.01, ***P<0.001. Error bars represent the S.E.M.

**
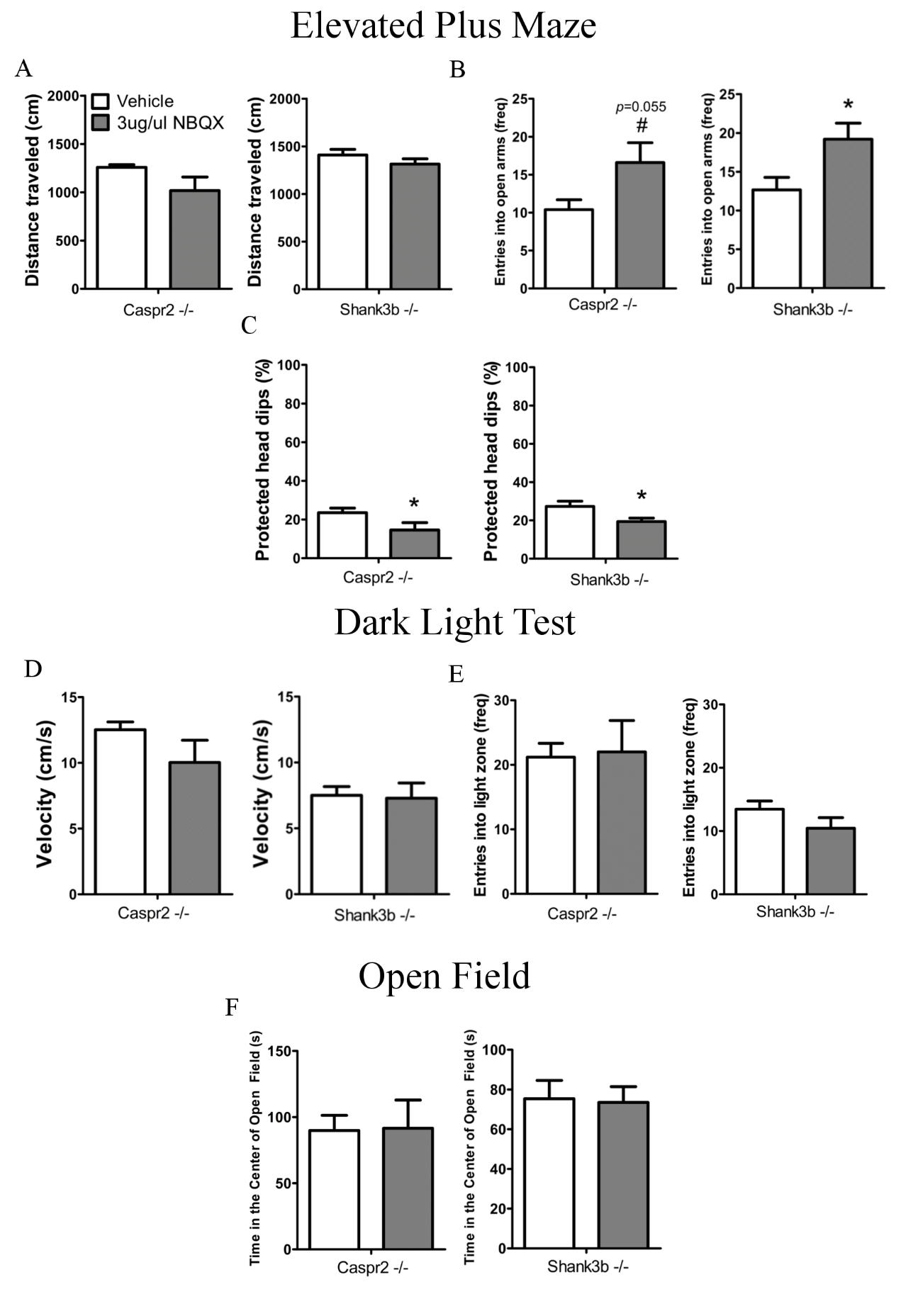
**

**Supplementary Figure S9 |** **Additional behavioral analysis of *CASPR2* KO and *SHANK3b* KO after treatment with NBQX or vehicle in the EPM, DL and OF.** (A) Total distance traveled in the EPM by *CASPR2* KO and *SHANK3b* KO mice after NBQX or vehicle treatment. (B) Entries into the open arms of the EPM by *CASPR2* KO and *SHANK3b* KO mice after NBQX or vehicle treatment. (C) Relative frequency of head dips in the risk-assessment zone of the EPM performed by *CASPR2* KO and *SHANK3b* KO mice after NBQX or vehicle treatment. (D) Velocity in the light zone of the DL by *CASPR2* KO and *SHANK3b* KO mice after NBQX or vehicle treatment. (E) Entries into the light zone of the DL by *CASPR2* KO and *SHANK3b* KO mice after NBQX or vehicle treatment. (F) Time spent in the center of the OF by *CASPR2* KO and *SHANK3b* KO mice after NBQX or vehicle treatment. C57BL/6J NBQX n=10-11, Vehicle n=10; *SHANK3b* KO NBQX n=9, Vehicle n=10. *CASPR2* KO NBQX n=10-9, Vehicle n=10. 0.05<#P<0.1, *P<0.05, **P<0.01, ***P<0.001. Error bars represent the S.E.M.

**Supplementary table 1. List of differentially expressed genes in the dorsal striatum of the *CASPR2* mouse model.** For each gene, in each sample (KO or WT) FPKM (Value 1 and 2), fold change and FDR corrected *p*-value is presented.

**Supplementary table 2. List of differentially expressed genes in the dorsal striatum of the *SHANK3b* mouse model.** For each gene, in each sample (KO or WT) FPKM (Value 1 and 2), fold change and FDR corrected *p*-value is presented.

**Supplementary table 3. List of differentially expressed genes in the dorsal striatum of the Chr16p11.2 mouse model.** For each gene, in each sample (KO or WT) FPKM (Value 1 and 2), fold change and FDR corrected *p*-value is presented.

**Supplementary table 4. List of differentially expressed genes in the dorsal striatum of the BTBR mouse model.** For each gene, in each sample (KO or WT) FPKM (Value 1 and 2), fold change and FDR corrected *p*-value is presented.

**Supplementary table 5. RTpcr primer sequence list.**

**Supplementary table 6. Weighted Gene Co-expression Network Analysis.** For each gene, Module Membership (MM) is presented per module (color name. e.g., MMsalmon). The closer the MM value is to 1, the stronger the gene expression's correlation is to the module's eigengene.
